# Prevalence of and factors associated with sarcopenia among multi-ethnic ambulatory older Asians with type 2 diabetes mellitus in a primary care setting

**DOI:** 10.1186/s12877-019-1137-8

**Published:** 2019-04-29

**Authors:** Foon Yin Fung, Yi Ling Eileen Koh, Rahul Malhotra, Truls Ostbye, Ping Yein Lee, Sazlina Shariff Ghazali, Ngiap Chuan Tan

**Affiliations:** 1 Duke NUS Medical School, 8 College Road, Singapore, 169857 Singapore; 2 SingHealth Polyclinics, 167 Jalan Bukit Merah Connection One Tower 5 #15-10, Singapore, 150167 Singapore; 3 Faculty of Medicine and Health Sciences, Universiti Putra Malaysia, 43400 Serdang, Selangor Darul Ehsan Malaysia

**Keywords:** Sarcopenia, Aging, Diabetes, Hip circumference

## Abstract

**Background:**

Sarcopenia is the age-related loss of muscle mass and function, which increases fall risks in older persons. Hyperglycemia relating to Type-2 Diabetes Mellitus (T2DM) is postulated to aggravate sarcopenia. This study aimed to determine the prevalence of sarcopenia among ambulatory community-dwelling older patients, aged 60–89 years, with T2DM in a primary care setting and to identify factors which mitigate sarcopenia.

**Methods:**

A total of 387 patients were recruited from a public primary care clinic in Singapore. Data on their socio-demography, clinical and functional status, levels of physical activity (International Physical Activity Questionnaire) and frailty status was collected. The Asian Working Group for Sarcopenia (AWGS) criteria were used to define sarcopenia based on muscle mass, grip strength and gait speed.

**Results:**

The study population comprised men (53%), Chinese (69%), mean age = 68.3 ± SD5.66 years, lived in public housing (90%), had hypertension (88%) and dyslipidemia (96%). Their mean muscle mass was 6.3 ± SD1.2 kg/m^2^; mean gait speed was 1.0 ± SD0.2 m/s and mean grip strength was 25.5 ± SD8.1 kg. Overall, 30% had pre-sarcopenia, 24% with sarcopenia and 4% with severe sarcopenia. Age (OR = 1.14; 95%CI = 1.09–1.20;*p* < 0.001), multi-morbidity (OR = 1.25;95%CI = 1.05–1.49;*p* = 0.011) diabetic nephropathy (OR = 2.50;95%CI = 1.35–5.13;*p* = 0.004), hip circumference (OR = 0.86;95%CI = 0.82–0.90;p < 0.001) and number of clinic visits in past 1 year (OR = 0.74; 95%CI = 0.59–0.92;*p* = 0.008) were associated with sarcopenia.

**Conclusions:**

Using AWGS criteria, 58% of older patients with T2DM had pre-sarcopenia and sarcopenia. Age, diabetic nephropathy, hip circumference, multi-morbidity and fewer clinic visits, but not a recent single HBA1c reading, were significantly associated with sarcopenia among patients with T2DM. A longitudinal relationship between clinic visits and sarcopenia should be further evaluated.

(250 words)

## Background

Sarcopenia is the age-related loss of muscle mass and muscle function. It is a newly recognized geriatric syndrome [1] associated with adverse health outcomes in older persons, such as functional decline, physical disability, frailty, increased fall risk, poorer quality of life, increased healthcare costs and higher mortality [2–4]. According to the diagnostic criteria defined by the Asian Working Group for Sarcopenia (AWGS), sarcopenia is diagnosed when there is low muscle mass (defined as skeletal muscle index < 7 kg/m^2^ in males and < 5.7 kg/m^2^ in females), together with either low muscle strength (defined as handgrip strength < 26 kg in males and < 18 kg in females) or low physical performance (defined as six-meter gait speed ≤0.8 m/s) or both [5]. Sarcopenia can be regarded as a spectrum of severity. As for the staging of sarcopenia, the European Working Group on Sarcopenia in Older People (EWGSOP), ‘pre-sarcopenia’ is characterized by low muscle mass without impact on muscle strength or physical performance; ‘sarcopenia’ is defined by two criteria: low muscle mass, plus low muscle strength or low physical performance; ‘severe sarcopenia’ is present when all three criteria of the definition are met [1].

The loss of muscle mass has been attributed to aging, underlying inflammation, endocrine dysfunction, insulin resistance, nutritional deficit and physical inactivity [1]. The interaction between genetics and environmental factors are likely to influence the risk of sarcopenia in different populations. Type 2 diabetes mellitus (T2DM) is a genetically-predisposed endocrine disorder resulting in insulin resistance and dysglycemia, which is aggravated by lifestyle behavioural factors such as dietary indiscretion and low physical activity. It is a burgeoning non-communicable disease (NCD) worldwide: 60% of the world’s population with T2DM is found in Asia [6]. Recent biochemical studies have shown growing evidence of an association between T2DM and sarcopenia. Intracellular insulin signaling cascade activates the mTOR pathway and inhibits autophagy, including lysosomal degradation of proteins and organelles, including those in the muscles. Insulin resistance due to T2DM may interfere with this signaling mechanism and contributes to accelerated muscle loss [7]. Suppressing insulin signaling also downregulates the phosphatidylinositol-3-kinase pathway, leading to decreased protein synthesis, which can be detrimental to muscle integrity and function [8]. With rising prevalence of T2DM globally, increasing number of older patients will be at risk of sarcopenia.

In Singapore, 11.3% of its adult population has T2DM, affecting a higher proportion of its minority Malay and Indian ethnic groups. The prevalence of T2DM is estimated to rise to 15% in 2050 [9]. Concurrently, WHO has identified Singapore as a nation with a population with the 3rd highest longevity in the world in 2018 [10]. Consequently, the prevalence of sarcopenia is expected to rise significantly due to its expanding aging population of 5.5 million, as those aged 65 years and above, is projected to more than doubled from 440,000 in 2015 to 900,000 by 2030 [11]. With a multi-ethnic Asian population with high prevalence of T2DM in the community, Singapore is a convenient test-bed to determine the epidemiology of sarcopenia among its ambulatory older Asian patients with T2DM. Their diverse backgrounds present opportunities to identify modifiable factors such as physical activity and glycemic control, which may retard the progression of sarcopenia.

Hence, this study primarily aimed to determine the prevalence of sarcopenia in ambulatory, older Asian patients with T2DM in a primary care setting in Singapore using the AWGS criteria. The secondary aim was to identify factors which may potentially mitigate sarcopenia risks in patients with T2DM. Understanding the magnitude of sarcopenia and associated mitigating factors in a vulnerable older population due to concurrent T2DM will influence the allocation of healthcare resources to scale up sarcopenia screening and facilitate the design of interventions to prevent further deterioration of muscle strength and function.

## Methods

This baseline study was conducted from October 2017 to March 2018 at a public primary care clinic (polyclinic) located within Pasir Ris estate in the north-eastern region of Singapore. The polyclinic serves 140,000 multi-ethnic Asian residents and manages about 570 patient attendances daily during office hours, of which 30% are aged 65 years and above. This study is part of a longitudinal study in which the study population will be reviewed 1 year later to re-assess for the development or progression of sarcopenia, to develop a predictive risk model of sarcopenia. In order to better quantify any progression 1 year later, we classified the participants into the different stages of sarcopenia according to the EWGSOP criteria, on top of using the AWGS diagnostic criteria, which did not include the different stages of sarcopenia.

### Study participants

The study population comprised multi-ethic Asian patients aged 60–89 years old, with a diagnosis of T2DM for at least 1 year from the data of their electronic medical records, regardless of their mode of therapeutic treatment. They were on regular medical reviews with two or more visits at the study site in the past 1 year. The participants can be treated with any therapeutic options compatible with their glycaemic control, ranging from diet control alone, oral hypoglycaemic agents alone, or a combination of oral hypoglycaemic agents with insulin injections.

Those with known risks which hindered or compounded sarcopenia assessment, such as history of stroke, carpal tunnel syndrome, severe hip or knee osteoarthritis, dysarthria or dysphasia, hearing difficulties, use of walking aid, physical disabilities that affect hand-grip and/or walking, use of electronic implants such as pacemaker, and living in residential care facilities were excluded. Patients with any form of other disabilities, such as cognitive impairment, which rendered them incapable of providing informed written consent were also excluded.

### Sample size estimation

The primary aim was to determine the prevalence of the sarcopenia in community-living, unassisted ambulatory T2DM primary care patients aged 60–89 years old. Utilizing the prevalence estimate (59.8%) for sarcopenia among older persons regardless of diabetes status from a recent Malaysian study [12], the sample size was computed to be 370, at 5% precision and 95% confidence level, using the following sample size formula: $n=\frac{Z^2P\left(1-P\right)}{d^2}$ where *Z* is *Z* statistic for a level of confidence, *P* is the expected prevalence and *d* is precision level. To account for 5% incomplete or missing data, the sample size was increased to 388. This was projected as a conservative estimate, as the prevalence was anticipated to be higher in the presence of T2DM.

### Recruitment and sarcopenia assessment

Case-encounter approach was used to recruit the potential participants. They were screened based on eligibility criteria at service points at the study site. They were provided the approved patient information sheet, clarified on their queries and recruited after the investigator obtained their informed written consent. Next, the subjects administered the study questionnaire, either by themselves or assisted by the investigator, to collect their demographic, lifestyle habits (alcohol intake, smoking status, and physical activity), socio-economic status and clinical information. Next, anthropometric assessment were performed to measure their weight, height, body mass index (BMI), waist circumference (WC), hip circumference (HC), systolic and diastolic blood pressures.

The participants stood erect on the calibrated AVAMECH Model B100U device, which measured their weight and height, automatically computed the BMI and printed out the parameters. The blood pressures were measured twice using the automatic blood pressure monitor (OMRON HEM-7280 T). WC was measured at the approximate midpoint between the lower margin of the last palpable rib and the top of the iliac crest while HC was measured around the widest portion of the pelvis [13]. Both WC and HC were determined using the same measuring tape.

Next, the sarcopenia assessment was performed:

1. body muscle mass was measured using a bio-electrical impedance analysis machine (OMRON Body composition monitor, Model HBF-375) according to the study protocol. The skeletal muscle index was then calculated as body muscle mass divided by squared body height.
2. handgrip strength was measured twice on each hand, using a dynamometer (JAMAR Plus Digital Hand Dynamometer #563213) with the subject seated with elbow flexed at ninety degrees, forearm in neutral position and wrist between 0 and 30 degrees of dorsiflexion and supported on a table, according to the American Society of Hand Therapists’ guidelines [14]. The average handgrip strength of the dominant hand was used for analysis;
3. six-meter gait speed was computed based on measurement of the average time taken for the subject to walk along a straight distance of six meters at usual walking speed. In this gait speed assessment, there were run-in and run-out phases of approximately 1 meter, before and after the six-meter distance respectively. Two time measurements were taken for each subject using a digital stopwatch (CASIO Model 611Q24R).

Sarcopenia was diagnosed according to the AWGS criteria [5] and further staged according to European Work Group for Sarcopenia (EWGSOP) guidelines [1]. Sarcopenia was diagnosed when there was low muscle mass (defined as skeletal muscle index < 7 kg/m^2^ in males and < 5.7 kg/m^2^ in females), together with either low muscle strength (defined as handgrip strength < 26 kg in males and < 18 kg in females) or low physical performance (defined as six-meter gait speed ≤0.8 m/s) or both.

The participants’ electronic medical records (EMR) were accessed to retrieve information on the latest glycemic control index (HbA1C) and fasting lipid profile (total cholesterol, high-density lipoprotein cholesterol [HDL], low-density lipoprotein cholesterol [LDL], triglycerides [TG]) from the laboratory test results. The duration of diabetes, presence of diabetic complications (any documented retinopathy, nephropathy, neuropathy, vasculopathy), co-morbidities (diagnosis list) and medications (electronic prescription) were also obtained. All data were audited and de-identified before being analyzed.

### Statistical analyses

Data were analyzed using the Statistical Package for Social science (SPSS) software (IBM, Version 21). Prevalence of sarcopenia (in stages) and categorical demographic and clinical variables were reported in frequencies and percentages. Muscle mass, handgrip strength, gait speed and continuous parameters were reported as mean ± standard deviation (SD). In the univariate and multivariate analyses, the outcome “Sarcopenia” is defined as those having sarcopenia and severe sarcopenia. No sarcopenia and pre sarcopenia were grouped as “No sarcopenia”. Univariate logistic regression analysis was performed to explore the factors associated with the presence of sarcopenia. Significant variables via backward selection approach were entered into the multivariable logistic regression to determine the factors associated with sarcopenia. Statistical significance was set at *P* ≤ 0.05.

### Ethics approval and funding

This study was reviewed and approved by the SingHealth Centralized Institutional Review Board (CIRB reference 2017/2393). This study was funded by a grant from Mitsui Sumitomo Insurance Welfare Foundation. The SingHealth-Duke NUS Academic Medicine Ethos Award supported the medical student (FFY) in the team and Omron Healthcare Singapore sponsored the Bio-impedance Assessment (BIA) device.

## Results

In total, 2056 patients were screened, of which 1483 failed the eligibility criteria, 183 declined study participation, 2 withdrew from the study, 1 disqualified after review of EMR due to exclusion criteria, and 387 patients with complete data were analyzed. The response rate was 67.7%. The demographic characteristics of the study population are summarized in Table 1. Their mean age was 68.3 ± 5.7 years. The duration of T2DM in the participants ranged from 1 to 50 years. The therapeutic interventions received by the participants included diet control alone, oral hypoglycemic agents alone, or a combination of oral hypoglycemic agents with insulin injections.

**Table 1:** Characteristics of the study population and their sarcopenia status

Characteristics^b^	Total *N* = 387	No Sarcopenia *n* = 163	Pre-sarcopenia *n* = 118	Sarcopenia *n* = 91	Severe sarcopenia *n* = 15
Age, years(mean/SD)	68.3(5.7)	66.5(4.4)	68.0(5.0)	70.4(6.2)	78.3(5.3)
60–69	245 (63.3)	129 (79.1)	73 (61.9)	42 (46.2)	1 (6.7)
70 and above	142 (36.7)	34 (20.9)	45 (38.1)	49 (53.8)	14 (93.3)
Gender					
Female	181 (46.8)	57 (35.0)	63 (53.4)	50 (54.9)	11 (73.3)
Male	206 (53.2)	106 (65.0)	55 (46.6)	41 (45.1)	4 (26.7)
Ethnicity					
Chinese	266 (68.7)	91 (55.8)	91 (77.1)	70 (76.9)	14 (93.3)
Malay	64 (16.5)	36 (22.1)	17 (14.4)	11 (12.1)	0 (0.0)
Indian	31 (8.0)	18 (11.0)	6 (5.1)	6 (6.6)	1 (3.2)
Others	26 (6.7)	18 (11.0)	4 (3.4)	4 (4.4)	0 (0.0)
Marital status					
Married	318 (82.2)	141 (86.5)	96 (81.4)	72 (79.1)	9 (60.0)
Single	15 (3.9)	6 (3.7)	5 (4.2)	4 (4.4)	0 (0.0)
Divorced/Separated	15 (3.9)	8 (4.9)	4 (3.4)	3 (3.3)	0 (0.0)
Widowed	39 (10.1)	8 (4.9)	13 (11.0)	12 (13.2)	6 (40.0)
Highest Qualification					
Up to primary education	92 (23.8)	29 (17.8)	31 (26.3)	22 (24.2)	10 (66.7)
Secondary education and beyond	295 (76.2)	134 (82.2)	87 (73.7)	69 (75.8)	5 (33.3)
Comorbidities					
Hypertension/ High blood pressure					
Yes	339 (87.6)	147 (90.2)	97 (82.2)	82 (90.1)	13 (86.7)
No	48 (12.4)	16 (9.8)	21 (17.8)	9 (9.9)	2 (13.3)
Hyperlipidemia/ High Cholesterol					
Yes	373 (96.4)	159 (97.5)	114 (96.6)	85 (93.4)	15 (100)
No	14 (3.6)	4 (2.5)	4 (3.4)	6 (6.6)	0 (0)
Ischemic Heart Disease					
Yes	74 (19.1)	32 (19.6)	15 (12.7)	20 (22)	7 (46.7)
No	313 (80.9)	131 (80.4)	103 (87.3)	71 (78)	8 (53.3)
Chronic kidney disease					
Yes	67 (17.3)	28 (17.2)	14 (11.9)	18 (19.8)	7 (46.7)
No	320 (82.7)	135 (82.8)	104 (88.1)	73 (80.2)	8 (53.3)
Anaemia					
Yes	31 (8)	10 (6.1)	6 (5.1)	10 (11)	5 (33.3)
No	356 (92)	153 (93.9)	112 (94.9)	81 (89)	10 (66.7)
^a^ Others					
Yes	240 (62.0)	100 (41.7)	63 (26.3)	64 (26.7)	13 (5.4)
No	147 (38.0)	63 (42.9)	55 (37.4)	27 (18.4)	2 (1.4)
Total number of medical conditions	4 (3–5)	4 (3–5)	4 (3–5)	4 (4–5)	6 (5–8)
Total number of long term medications	6 (4–8)	6 (5–8)	5.5 (4–7)	6 (5–8)	7 (6–8)
Type of dwelling					
Public housing (Rental flat/1-3room)	54 (14.0)	19 (11.7)	16 (13.6)	17 (18.7)	2 (13.3)
Public housing (4–5 room)	287 (74.2)	129 (79.1)	84 (71.2)	65 (71.4)	9 (60.0)
Condominium/Private property	46 (11.9)	15 (9.2)	18 (15.3)	9 (9.9)	4 (26.7)
Living					
Alone	16 (4.1)	5 (3.1)	6 (5.1)	4 (4.4)	1 (6.7)
With family	367 (96.8)	156 (95.7)	111 (94.1)	87 (95.6)	13 (86.7)
With assistance from domestic helper	4 (1.0)	2 (1.2)	1 (0.8)	0 (0.0)	1 (6.7)
Medical subsidy					
Public assistance	106 (27.4)	49 (30.1)	29 (24.6)	24 (26.4)	4 (26.7)
Waiver of medical fee	0 (0.0)	0 (0.0)	0 (0.0)	0 (0.0)	0 (0.0)

^a^Others include Asthma, Cateract, Osteoarthritis, Gout etc

^b^Continuous variables reported as mean ± SD; Categorical variables reported as frequency (%)

**Fig. 1:**
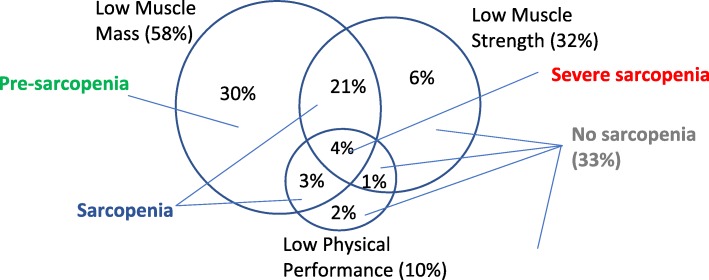
Venn diagram showing Proportions of Patients with Low Muscle Mass, Low Muscle Strength and Low Physical Performance

The overall prevalence of sarcopenia among the community-living, unassisted ambulatory patients aged 60–89 years with T2DM was 27.4%, among which 3.9% had severe sarcopenia and 30.5% had pre-sarcopenia (Fig. 1). The proportion with low muscle mass, low muscle strength and low gait speed were 57.9, 31.3 and 9.6% respectively (Fig. 1).

The factors associated with sarcopenia are summarized in Table 2. Univariate logistic regression analyses showed that demographic factors, such as age (OR = 1.15, 95%CI = 1.10–1.20, *p* < 0.001), women (OR = 1.82, 95%CI = 1.16–2.86, *p* = 0.009) and Chinese ethnicity (OR = 2.08, 95%CI = 1.16–2.86, *p* = 0.007) were associated with greater risk of sarcopenia. However, multivariable logistic regression analyses revealed that only age remained as a significant demographic factor (Table 3).

**Table 2:** Factors associated with sarcopenia using univariate analysis

Characteristic	Crude Odds Ratio (95% CI)	*P* value
Age, years	1.15 (1.10–1.20)	< 0.001
Gender		
Men	1	–
Women	1.82 (1.16–2.86)	0.009
Ethnicity		
Malay/ Indian/ Others	1	–
Chinese	2.08 (1.22–3.53)	0.007
Physical Activity level		
Done light house work in past 1 week		
No	1	
Yes	1.75 (1.02–3.03)	0.043
Done heavy house work in past 1 week		
No	1	
Yes	1.45 (0.93–2.27)	0.105
Worked for pay/volunteer in past 1 week		
No	1	
Yes	2.00 (1.25–3.13)	0.004
Vigorous activity, min/day	0.95 (0.87–1.04)	0.292
Moderate activity, min/day	1.01 (1.00–1.01)	0.685
Walking, min/day	0.99 (0.99–1.00)	0.468
Sitting, min/day	0.99 (0.99–0.99)	0.005
Dietary protein intake		
Legumes/Lentils, number of meals	0.58 (0.37–0.91)	0.018
Meat/Seafood/Eggs, number of meals	1.40 (1.01–1.93)	0.041
Nuts/Soy, number of meals	1.16 (0.86–1.57)	0.321
Dairy products, number of meals	1.25 (0.92–1.68)	0.148
Other sources of protein, number of meals	0.66 (0.07–5.97)	0.711
Social demographics		
Married		
Yes	1	
No	1.67 (0.95–2.86)	0.071
Highest qualification (Secondary level & above)		
No	1	
Yes	0.63 (0.38–1.04)	0.07
Medical subsidy (CHAS card holder)		
No	1	
Yes	0.93 (0.56–1.55)	0.792
Lifestyle factors		
Current smoker		
No	1	
Yes	0.44 (0.15–1.30)	0.138
Regular alcohol use		
No	1	
Yes	0.43 (0.10–1.96)	0.276
Use of supplements/ complementary medicines		
No	1	
Yes	1.34 (0.86–2.10)	0.199
BMI, kg/m^2^	0.76 (0.70–0.83)	< 0.001
Waist Circumference, cm	0.92 (0.90–0.95)	< 0.001
Hip Circumference, cm	0.87 (0.83–0.90)	< 0.001
Waist/hip ratio	0.08 (0.00–4.77)	0.224
Systolic BP, mmHg	1.01 (1.00–1.02)	0.084
Diastolic BP, mmHg	0.98 (0.95–1.00)	0.042
HbA1C, %	0.81 (0.63–1.04)	0.093
Years of Diabetes	1.04 (1.01–1.07)	0.013
Diabetic nephropathy		
No	1	
Yes	1.99 (1.16–3.41)	0.013
Lipid profile		
Total Cholesterol, mmol/L	0.89 (0.67–1.19)	0.444
HDL, mmol/L	1.76 (0.95–3.26)	0.07
LDL, mmol/L	0.78 (0.54–1.12)	0.18
TG, mmol/L	0.79 (0.55–1.14)	0.21
Number of polyclinic visits with doctors last year	0.78 (0.64–0.95)	0.013
Chronic illnesses		
Chronic Kidney Disease		
No	1	–
Yes	1.76 (1.01–3.06)	0.047
Anaemia		
No	1	–
Yes	2.73 (1.30–5.74)	0.008
Hypertension		
No	1	–
Yes	1.31 (0.64–2.67)	0.459
Hyperlipidemia		
No	1	–
Yes	0.49 (0.17–1.44)	0.195
Ischemic Heart Disease		
No	1	–
Yes	1.70 (0.99–2.91)	0.053
Total number of medical conditions	1.22 (1.06–1.39)	0.005
Total number of long term medications	1.03 (0.94–1.12)	0.566

**Table 3:** Factors associated with sarcopenia using backward stepwise logistic regression

Characteristics	Chi-square	R-square	Adjusted Odds Ratio (95% CI)	*P* value
	122.520	0.393		
Age, years			1.14 (1.09–1.20)	< 0.001
Dietary protein intake				
Legumes/Lentils, number of meals			0.6 (0.35–1.04)	0.067
Hip Circumference, cm			0.86 (0.82–0.9)	< 0.001
Diabetic nephropathy				
No			1	–
Yes			2.50 (1.30–5.00)	0.006
Number of consultations at polyclinics in past 1 year			0.74 (0.59–0.92)	0.008
Number of medical conditions (multiple morbidities)			1.25 (1.05–1.49)	0.011

Engaging in physical activities such as having done light house work (OR = 1.75, 95%CI = 1.02–3.03, *p* = 0.043) and having worked for pay/volunteer (OR = 2.00; 95%CI = 1.25–3.13, *p* = 0.004) are associated with higher risk of sarcopenia, while sitting (OR = 0.998, 95%CI = 0.997–0.999; *p* = 0.005) was associated with lower risk of sarcopenia. Smoking and alcohol consumption were not associated with sarcopenia.

For dietary habits, univariate analysis found that consumption of legumes/lentils (OR = 0.579, 95%CI = 0.37–0.91, *p* = 0.018) was associated with reduced risk of sarcopenia. In contrast, consumption of meat/seafood/eggs (OR = 1.40, 95%CI = 1.01–1.93, *p* = 0.041) was associated with higher risk of sarcopenia. However, the physical activity and dietary factors were not significantly associated with sarcopenia after multivariable analyses.

The number of medical conditions or comorbidities (OR = 1.22; 95%CI = 1.06–1.39, p = 0.005), history of chronic kidney disease (OR = 1.76, 95%CI = 1.01–3.06, *p* = 0.013), anemia (OR = 2.73; 95%CI = 1.30–5.74, *p* = 0.008) and the number of polyclinic visits for consultation over the past year (OR = 0.78, 95%CI = 0.64–0.95, p = 0.013) were associated with sarcopenia in univariate analyses. Multivariate analyses showed that multiple morbidities and number of consultations at polyclinics in past year were significantly associated with sarcopenia.

Clinical parameter such as diastolic blood pressure (OR = 0.98, 95%CI = 0.95–1.99, *p* = 0.042) and anthropometric measurements such as BMI (OR = 0.76, 95%CI = 0.70–0.83, *p* < 0.001), WC (OR = 0.92, 95%CI = 0.90–0.95; p < 0.001) and HC (OR = 0.87, 95%CI = 0.83–0.90; p < 0.001) were associated with lower risk of sarcopenia. Only HC remained a significant factor after multivariable analyses.

The recent glycemic control index (up to 6 months ago), HbA1C (OR = 0.81, 95%CI = 0.63–1.04, *p* = 0.093) and lipid profiles were not associated with sarcopenia. Duration of T2DM (OR = 1.04, 95%CI = 1.01–1.07, *p* = 0.013) and the presence of diabetic nephropathy based on laboratory investigation (OR = 1.99, 95%CI = 1.16–3.41, p = 0.013) were identified as significant risk factors but only the latter remained associated with sarcopenia after multivariate analyses (OR = 2.50, 95%CI = 1.30–5.00, *p* = 0.006).

In summary (Table 3), advanced age, hip circumference, diabetic nephropathy, number of consultations at polyclinics and number of medical conditions (multiple morbidities) were associated with sarcopenia. The association between recent glycemic control index (HbA1C) and sarcopenia was not established in this study.

## Discussion

This study found that the prevalence of sarcopenia in older, community-dwelling patients with T2DM in Singapore was 27.4%. It seems lower compared with a Malaysian study (59.8%) by Norshafarina et al. with a similar multi-ethnic Asian study population [12]. However, Norshafarina et al. applied the EWGS diagnostic criteria and cut-off values for sarcopenia instead of those recommended by AWGS [12].

In comparison, the Korean and Japanese studies reported lower sarcopenia prevalence of 15.7 and 13.3%, respectively [15, 16]. Like Singapore, these are developed countries with similar socio-economic background as well as comparable healthy life expectancy after 60 years of age, ranging from 20.2 years in Singapore to 20 years in Korea and 21.1 years in Japan (http://www.who.int/gho/mortality_burden_disease/life_tables/situation_trends/en/). The variation between these findings may be attributed to different measurement methods and/or diagnostic criteria. Both the Korean and Japanese studies used dual-energy X-ray absorptiometry (DEXA) to quantify muscle mass, whereas this study used bio-electrical impedance analysis for the measurement. Furthermore, different definitions of low muscle mass and cut-off values were used in the Korean study [15]. Despite using the AWGS criteria, only muscle mass and muscle strength were measured in the Japanese study [16]. The gait speed assessment was omitted, creating challenges in comparison of sarcopenia prevalence between study populations.

The study baseline data did not show a similar correlation between the glycemic index and sarcopenia. It differs from the report by Yoon JW [17] that poor glycemic control (HbA1C above 8.5%) which showed a relationship between sarcopenia and decreased muscle performance status in their longitudinal Korean study. A single glycated hemoglobin index reflects the glycemic control over 3 months, which is probably too short to impact on the development of sarcopenia. This cohort of older patients is currently being monitored for their muscle mass and function over time, which will better allude to the effect of glycaemia on sarcopenia.

Advanced age and multiple morbidities were associated with higher risk of sarcopenia, which are compatible with the findings from previous studies [18–21]. The presence of diabetic nephropathy increased the risk of sarcopenia, which was likely to the direct chronic loss of proteins in the urine in diabetic nephropathy, resulting in reduced body muscle mass. Sarcopenia has previously been reported to be associated with declining renal function, leading to lower glomerular filtration rate and higher urine albumin to creatinine ratio [22].

What is more remarkable is that the number of polyclinic visits to consult doctors over the past year was associated with a lower risk of sarcopenia. This observation could be attributed to the health status and health-seeking behavior of the patients. Either they were physically stronger and functionally well, and hence were capable of travelling to the clinic more frequently, or they could be more health-conscious and tended to follow-up with their physicians more closely to monitor their health. On the other hand, those with overall poorer health, multiple morbidities and complications such as chronic kidney disease, will also require more frequent consultations. Further longitudinal study is needed to gain a better understanding of this associated factor.

Hip circumference (HC) assesses skeletal frame size, adipose and muscle mass in the buttock and thigh regions [23]. Higher HC suggests the presence of larger muscle mass in the gluteal area of the persons. This could explain the finding that higher HC was associated with lower sarcopenia.

The body muscle mass measured in this study is used as a proxy for appendicular skeletal mass. While this body muscle mass is not universally accepted in the assessment of sarcopenia, and potentially constitutes a study limitation, it is easier for implementation in ambulatory primary care or community setting using the potable bio-impedance analysis device. This is in contrast to the use of the Dual-energy X-Ray Absorptiometry (DXA), often in secondary or tertiary care setting due to the radiation, which requires computation to approximate the appendicular skeletal mass.

This study has several other limitations. The causal and chronological relationship of the associated factors with sarcopenia cannot be established from this cross-sectional study. For instance, while less physical activity maybe associated with higher risk of sarcopenia, sarcopenia could also result in reduced physical activity. The potential recall bias, as well as the data reliability and accuracy cannot be objectively ascertained in the self-reported variables. As patients with cognitive impairment or significant physical disabilities and/or pacemakers and dependent on walking aids were excluded, the findings are not generalizable to the wider, heterogeneous population of older patients with T2DM in Singapore. The data from a single study site would not reflect of the nation-wide prevalence of sarcopenia.

## Conclusion

Among every three community-dwelling, unassisted ambulatory older patients aged 60–89 years with T2DM in Singapore, nearly one had sarcopenia and one had pre-sarcopenia. Sarcopenia was significantly associated with advanced age, multiple morbidities, diabetic nephropathy, hip circumference and number of consultations at primary care clinics. A single recent glycaemic control index, HbA1C was not significantly associated with sarcopenia. A longitudinal relationship between clinic visits and sarcopenia should be further evaluated. Identifying the associated risk factors from this study may enable stratification of resource allocation for sarcopenia screening and intervention in this vulnerable group of older patients with T2DM.

## Abbreviations

AWGS: Asian Working Group for Sarcopenia
BMI: Body mass index
CI: Confidence interval
CIRB: Centralized Institutional Review Board
DEXA: Dual-energy X-ray absorptiometry
EMR: Electronic medical records
EWGSOP: European Working Group on Sarcopenia in Older People
HBA1c: Hemoglobin A1c/ glycated hemoglobin
HC: Hip circumference
HDL: High-density lipoprotein cholesterol
LDL: Low-density lipoprotein cholesterol
NCD: Non-communicable disease
NEFA: Non-esterified fatty acids
OR: Odds ratio
SD: Standard deviation
SPSS: Statistical Package for Social Science
T2DM: Type 2 diabetes mellitus
TG: Triglycerides
WC: Waist circumference
WHO: World Health Organization
